# Monocyte–plasma cell communication characterizes treatment-associated peripheral immune remodeling in Guillain–Barré syndrome

**DOI:** 10.3389/fimmu.2026.1931955

**Published:** 2026-08-13

**Authors:** Sumin Kim, Myung Ah Lee, Jaehyuk Jang, Minki Kim, Bo Kyung Yoon, Sungsoon Fang, Hyo-Suk Ahn, Nahee Hwang

**Affiliations:** 1 Department of Microbiology and Immunology, Institute for Immunology and Immunological Diseases, Yonsei University College of Medicine, Seoul, Republic of Korea; 2 Graduate School of Medical Science, Brain Korea 21 Project, Yonsei University College of Medicine, Seoul, Republic of Korea; 3 Department of Neurology, Uijeongbu St. Mary’s Hospital, College of Medicine, The Catholic University of Korea, Seoul, Republic of Korea; 4 Division of Cardiology, Department of Internal Medicine, Uijeongbu St Mary’s Hospital, College of Medicine, The Catholic University of Korea, Seoul, Republic of Korea; 5 Catholic Research Institute for Intractable Cardiovascular Disease, College of Medicine, The Catholic University of Korea, Seoul, Republic of Korea; 6 Center for Cellular Therapies and Cancer Immunology, Simmons Comprehensive Cancer Center, University of Texas Southwestern, Dallas, TX, United States; 7 Institute for Immunology and Immunological Diseases, Yonsei University College of Medicine, Seoul, Republic of Korea; 8 Department of Biomedical Sciences, Gangnam Severance Hospital, Yonsei University College of Medicine, Seoul, Republic of Korea

**Keywords:** autoimmune disease, B cell, double negative T cell, Guillain-Barré syndrome, monocyte, plasma cell

## Abstract

**Background:**

Guillain -Barré syndrome (GBS) is an acute immune-mediated polyradiculoneuropathy involving multifaceted pathogenic mechanisms. Although humoral immunity is central to GBS pathogenesis, longitudinal changes in peripheral immune circuits across symptomatic disease and clinical recovery remain incompletely understood at single-cell resolution.

**Methods:**

We performed longitudinal single-cell RNA sequencing of peripheral blood mononuclear cells collected during the symptomatic GBS and clinical recovery phases from a patient with GBS. Changes in immune cell composition, transcriptional programs, intercellular communication, and gene regulatory networks were examined, with particular attention to plasma cells, monocytes, B cells, and putative double-negative T cells (DNTs).

**Results:**

Plasma cells, monocytes, and putative DNTs exhibited stage-dependent transcriptional remodeling. During the GBS phase, plasma cells exhibited increased secretory and IgG-associated programs, consistent with enhanced antibody-producing activity. Monocytes represented higher relative proportion and showed enrichment of antigen-presentation and inflammatory pathways. Cell -cell communication analysis indicated stronger inferred BAFF and APRIL signaling from monocytes toward B cells and plasma cells during the GBS phase, suggesting a potential monocyte contribution to humoral activation. Regulatory network analysis identified SPI1-associated activity within the inflammatory monocyte program. In parallel, putative DNTs showed comparatively higher inferred outgoing cytokine signaling together with enrichment of ribosome biogenesis and protein synthesis pathways. During recovery, plasma cell secretory signatures were attenuated. B cells upregulated memory- and regulatory-associated genes, inflammatory monocyte programs diminished, and inferred DNT-associated cytokine signaling was lower in the recovery sample, accompanied by receptor-mediated and adhesion-related transcriptional programs.

**Conclusion:**

Collectively, these findings provide a longitudinal single-cell view of peripheral immune remodeling in GBS and highlight a stage-associated monocyte -B-cell regulatory axis and differences in DNT-associated inferred cytokine signaling between the GBS and recovery samples.

## Introduction

Guillain–Barré syndrome (GBS) is an acute immune-mediated polyradiculoneuropathy characterized by rapidly progressive limb weakness and decreased or absent deep tendon reflexes. Its clinical manifestations range from mild sensory disturbances to flaccid paralysis, respiratory failure, and autonomic dysfunction (1). While the overall prognosis for GBS is favorable, mortality still occurs in 3–10% of cases, and it has been reported that as many as 20% to 35% of patients suffer from persistent neurological disability (2). This clinical heterogeneity, together with the lack of reliable biomarkers for predicting disease progression and recovery, highlights the need to define the peripheral immune mechanisms that shape GBS severity and treatment response.

Although GBS is classified as a rare disease, some studies have reported an increasing trend in its global incidence in recent decades. Population aging, evolving infectious exposures, and outbreaks of pathogens capable of triggering neuroimmune responses—such as Campylobacter jejuni, Zika virus, and SARS-CoV-2—are thought to contribute to this trend (3, 4). Transient surges in GBS incidence following infectious epidemics further highlight its epidemiological relevance and reinforce the importance of understanding the systemic immune processes that shape disease variability and treatment responsiveness.

Although GBS manifests clinically within the peripheral nervous system, its pathogenesis involves broader systemic immune dysregulation. Post-infectious innate immune activation, anti-ganglioside antibody production, and dysregulated T cell–macrophage interactions collectively contribute to peripheral nerve injury (5). These immune perturbations may be captured in circulating immune populations, positioning peripheral blood mononuclear cells (PBMCs) as an accessible window into disease activity and immune network dynamics.

Among circulating immune subsets, monocytes are of particular interest in GBS because they can give rise to macrophage populations involved in myelin and axonal injury and regulate the magnitude and quality of acute inflammatory responses (6, 7). Their transcriptional states may therefore reflect disease activity, treatment responsiveness, and recovery trajectories, supporting their potential utility as dynamic immune biomarkers. However, how monocyte subpopulations and their associated signaling networks are reorganized during treatment-associated recovery remains insufficiently defined at single-cell resolution.

Standard therapies for GBS, including intravenous immunoglobulin (IVIg) and plasma exchange (PE), target circulating immune components. IVIg exerts broad immunomodulatory effects, including modulation of pathogenic antibodies, Fc receptor signaling, and complement activation, whereas PE removes autoantibodies, immune complexes, cytokines, and other inflammatory mediators (8, 9). Despite their clinical efficacy, how these interventions reshape immune cell composition, transcriptional states, and intercellular communication networks remains poorly defined. This gap highlights the value of longitudinal PBMC profiling for defining treatment-associated systemic immune remodeling.

Recent single-cell RNA sequencing (scRNA-seq) studies have identified acute myeloid activation in GBS, including disease-associated monocyte subsets and T cell–macrophage inflammatory signaling (10, 11). However, these studies were primarily cross-sectional and did not define how myeloid, humoral, and lymphoid immune programs are coordinately remodeled within the same patient over time. Paired longitudinal analyses across the symptomatic GBS and recovery phases are therefore needed to clarify stage-associated immune network remodeling.

Here, using paired PBMC samples collected at two clinically distinct time points, we characterized temporal changes in plasma cell secretory activity, monocyte-derived BAFF/APRIL signaling toward B cells and plasma cells, SPI1-associated myeloid regulation, and DNT-associated inferred cytokine signaling. This framework enabled us to nominate candidate regulatory axes associated with treatment-related immune reorganization in GBS.

## Methods

### Clinical information

61-year-old male patient with clinically diagnosed Guillain–Barré syndrome (GBS) was enrolled for longitudinal PBMC single-cell RNA sequencing. Neurological symptoms began on August 15, 2023, following preceding upper respiratory symptoms. The patient reached clinical nadir approximately between August 20 and 24, 2023, with generalized weakness and areflexia accompanied by respiratory and swallowing dysfunction requiring intubation and mechanical ventilation. IVIg was administered from August 18 to 22, 2023, at a total dose of 2 g/kg over 5 days. Electrophysiological evaluation supported the acute inflammatory demyelinating polyneuropathy subtype. Cerebrospinal fluid analysis showed 2 white blood cells/µL and a protein concentration of 29 mg/dL, without albuminocytologic dissociation. Anti-ganglioside antibody testing was negative.

The first PBMC sample, designated the GBS sample, was collected on September 5, 2023, 21 days after symptom onset and 14 days after completion of IVIg. At this time, residual generalized weakness and discomfort remained, although the patient was independently ambulatory and breathing spontaneously. The follow-up PBMC sample, designated the recovery sample, was collected on November 22, 2023, 99 days after symptom onset, 92 days after completion of IVIg, and 78 days after the first sampling. At the follow-up time point, the patient was independently ambulatory and breathing spontaneously with clinical recovery.

### PBMC isolation

PBMCs were isolated on the same day as blood collection. Whole blood collected in EDTA tubes was diluted 1:1 with phosphate-buffered saline (PBS) and transferred to Leucosep tubes. Samples were centrifuged at 1000 × g for 15 min at room temperature. The mononuclear cell layer was collected and centrifuged at 400 × g for 10 min. After removal of the supernatant, cells were washed twice and counted. Cells were resuspended in freezing medium consisting of 10% dimethyl sulfoxide (DMSO) in fetal bovine serum (FBS), stored at −80 °C for 24 h, and subsequently transferred to liquid nitrogen for long-term storage.

### Chromium next GEM single cell 3′ v3.1 library preparation

Cell viability was assessed using a LUNA-FL™ automated fluorescence cell counter (Logos Biosystems, Republic of Korea) according to the 10x Genomics Single Cell Preparation Guide. Cell viability was 92% and 96% for the GBS and recovery samples, respectively. Single-cell RNA-sequencing libraries were generated using the Chromium Next GEM Single Cell 3′ RNA library v3.1 platform (10x Genomics, Pleasanton, CA, USA). Approximately 10,000 cells were targeted for recovery per sample. Cell suspensions were loaded onto a Chromium chip to generate Gel Bead-In Emulsions (GEMs), in which cellular transcripts were barcoded and reverse-transcribed. Following cDNA amplification, sequencing libraries were prepared according to the manufacturer’s protocol. Library quality was evaluated using an Agilent TapeStation, and library concentrations were quantified by qPCR. Libraries were sequenced on an Illumina platform using paired-end sequencing.

### Single cell RNA-seq

Raw base-call files were demultiplexed into FASTQ files using the cellranger mkfastq pipeline in Cell Ranger v8.0.1, with bcl2fastq v2.20.0. Sequencing reads were aligned to the human reference genome GRCh38-2024-A using the cellranger count pipeline v8.0.1 (10x Genomics). Cell Ranger was used for read alignment, barcode filtering, UMI counting, and generation of feature-barcode matrices.

### Quality control, data integration, and unsupervised clustering

Single-cell RNA-seq data were analyzed using Seurat v5.4.0 in R (12). Cells with more than 200 and fewer than 6,000 detected genes and a mitochondrial transcript proportion below 10% were retained. No explicit filtering threshold based on nCount_RNA was applied. Doublets were identified using scDblFinder and excluded from downstream analyses. The predicted doublet rates were 10.5% and 16.9% for the GBS and recovery samples, respectively. After quality-control filtering and doublet removal, 12,192 and 15,298 singlets were retained from the GBS and recovery samples, respectively, yielding a total of 27,490 cells for downstream analysis. Ambient RNA correction was not performed. Per-sample viability, sequencing depth, median genes and UMIs per cell, final retained cell numbers, and cell numbers for each annotated population are provided in **Supplementary Table 1**. Each sample was normalized using NormalizeData, followed by variable-feature selection using FindVariableFeatures. Integration anchors were identified using FindIntegrationAnchors, and the samples were integrated using IntegrateData. The integrated data were subsequently scaled and subjected to principal component analysis, graph-based clustering, and UMAP visualization. Dimensionality reduction, graph-based clustering, differential gene expression analysis, and cell-type annotation were performed according to established marker genes. Sixteen transcriptionally distinct clusters were identified, including naïve T cells, putative double-negative T cells (DNTs), CD8^+^ T cells, cytotoxic CD8^+^ T cells, MAIT cells, CD4^+^ T cells, regulatory T cells, γδ T cells, natural killer (NK) cells, B cells, plasma cells, monocytes, megakaryocytes, and a small population of unclassified cells. The DNT population was identified based on its transcriptomic profile and is therefore referred to as a putative DNT population in the absence of flow-cytometric confirmation.

### Differential gene expression and functional analysis

Differentially expressed genes (DEGs) were identified using the FindAllMarkers function in Seurat to characterize the transcriptional features of each cell subcluster. Statistical testing was performed using the Wilcoxon rank-sum test on normalized expression values from the RNA assay. Genes were classified as differentially expressed when they had a Bonferroni-adjusted P value (Seurat p_val_adj) < 0.05, were detected in at least 25% of cells in either comparison group, and had an absolute average log2 fold change (avg_log2FC) > 0.58. These three criteria were applied jointly using an AND condition. Because the initial significance- and detection-based filtering identified a large number of DEGs, the more stringent absolute log2 fold-change threshold of 0.58 was applied to prioritize genes with more pronounced expression differences for visualization and downstream functional interpretation.

Functional enrichment analysis was performed using clusterProfiler v4.14.6 (13) to identify biological processes represented by the resulting DEG sets. Gene Ontology over-representation analysis was conducted using terms from the Biological Process category. In addition, pathway- and cell-state-associated signature scores were calculated using curated gene sets from the ESCAPE database and its accompanying R package (14). Enrichment and signature-analysis results were visualized using violin plots and enrichment network maps.

Because this study included paired samples from a single patient, the patient, rather than the individual cell, was considered the biological unit. Individual cells were not considered independent biological replicates. Accordingly, Wilcoxon tests were used only for exploratory cell-level comparisons, and the resulting P values were not interpreted as patient-level statistical inference or evidence of biological reproducibility.

### Cell-cell communication analysis

Cell–cell communication among annotated cell clusters was inferred using the CellChat R package v2.2.0, which models intercellular signaling based on curated ligand–receptor interactions (15). Communication probabilities were calculated using the computeCommunProb function, with average gene expression within each cluster summarized by the trimean method.

To characterize the signaling roles of individual cell populations, incoming and outgoing communication strengths were visualized using the netAnalysis_signalingRole_scatter function. Communication probabilities were calculated separately for the GBS and recovery samples using the trimean method with population.size = FALSE, such that inferred communication strength was not directly weighted by the relative abundance of each cell population. CellChat P values reflect the package’s internal permutation-based assessment of individual ligand–receptor interactions within each sample and do not test differences between the two time points. Pathway-level information flow was therefore compared descriptively using rankNet. Raw cell numbers for each annotated population and the CellChat minimum-cell and ligand–receptor filtering parameters are provided in **Supplementary Table 1**.

### Regulon activity analysis

Transcription factor activity was inferred using the SCENIC pipeline v1.3.1 in R (16). Gene regulatory networks were reconstructed from the single-cell transcriptomic data using GENIE3, followed by motif-enrichment analysis with RcisTarget to identify putative direct transcription factor–target gene interactions. Regulon activity was quantified at the single-cell level using AUCell.

For the complementary iRegulon analysis, differentially expressed genes between monocytes and comparator cell populations were identified separately in the GBS and recovery samples using Seurat. Genes with an average log2 fold change (avg_log2FC) > 0.25 and a Bonferroni-adjusted P value (Seurat p_val_adj) < 0.05 were considered upregulated and were used as input for iRegulon analysis in Cytoscape to identify candidate upstream transcriptional regulators (17, 18). Transcription factors associated with BAFF/APRIL signaling components were further prioritized for downstream analysis.

A lower log2 fold-change threshold of 0.25 was used for the iRegulon analysis to retain a sufficiently broad set of directionally upregulated candidate genes for motif-enrichment and transcription-factor prediction. In contrast, the more stringent absolute log2 fold-change threshold of 0.58 was used in the primary DEG analysis to reduce the large number of statistically significant genes and prioritize more pronounced transcriptional differences for visualization and functional interpretation. Thus, the two thresholds were selected according to the distinct purposes of the respective analyses.

## Results

### Result 1. Single-cell profiling reveals stage-dependent remodeling of circulating immune cells in GBS

**Figure 1:**
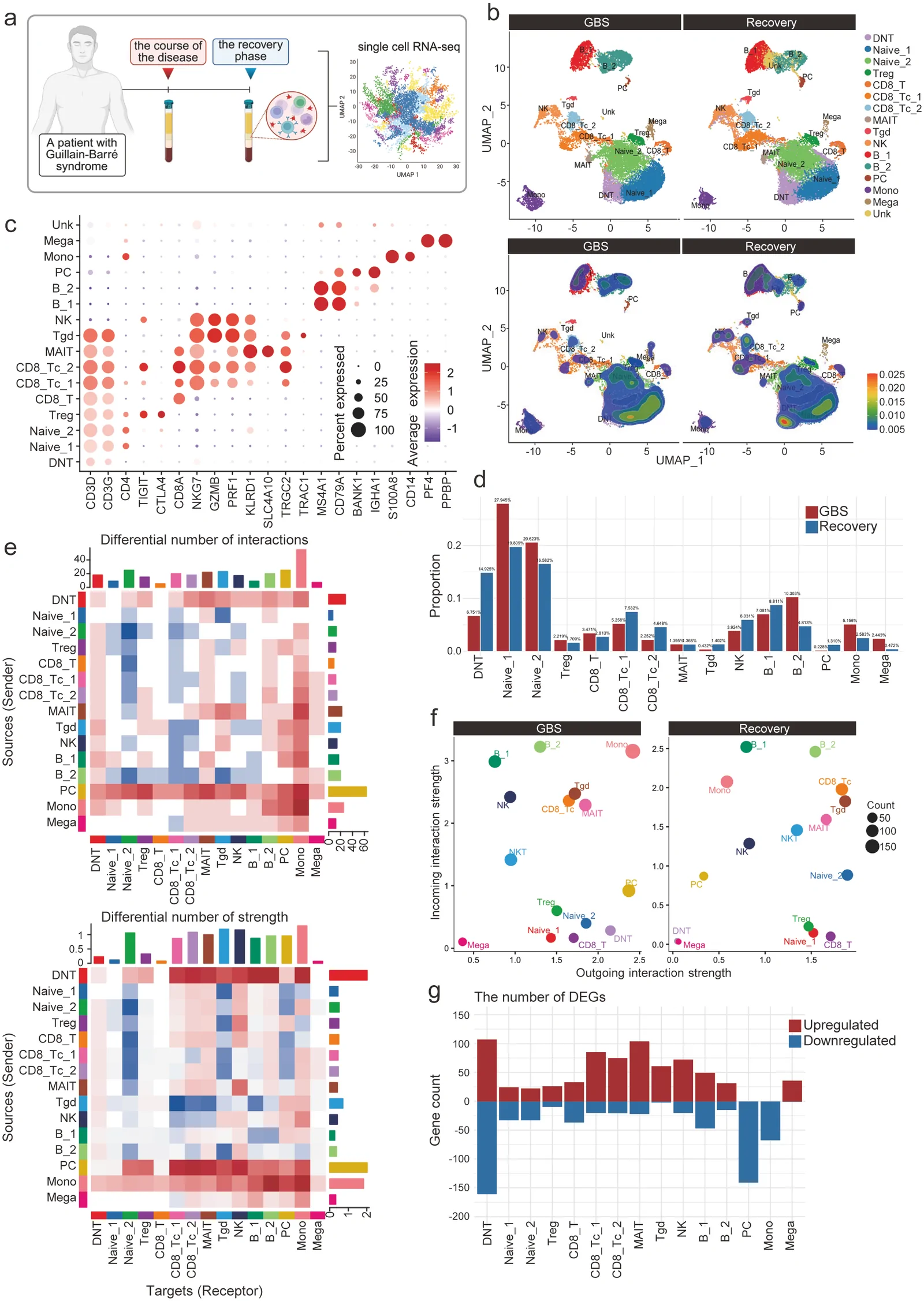
Single-cell profiling reveals stage-dependent changes in PBMC composition and intercellular signaling in GBS. **(A)** Schematic overview of the single-cell RNA sequencing workflow applied to PBMC samples collected from a Guillain–Barré syndrome (GBS) patient during symptomatic GBS and clinical recovery phases. **(B)** UMAP visualization of 16 distinct immune cell clusters identified from PBMCs. Density contours below indicate cluster-specific cell distributions. **(C)** Dot plot showing canonical marker gene expression used for cell-type annotation. **(D)** Bar plot depicting stage-dependent changes in cell-type proportions between the GBS and recovery phases. **(E)** Heatmaps illustrating ligand–receptor interaction strength and frequency across cell subtypes in the GBS phase. **(F)** Scatter plots identifying dominant sender and receiver cell populations in cytokine-mediated communication networks, comparing GBS and recovery phases. **(G)** Bar plot showing the number of differentially expressed genes (DEGs) in each cell subtype during the GBS phase. For **(E, F)**, CellChat comparisons represent descriptive within-patient observations, and no patient-level inferential statistical test was performed. For panel g, DEGs were identified using exploratory cell-level Wilcoxon comparisons, and individual cells were not considered independent biological replicates.

Peripheral blood mononuclear cells (PBMCs) were collected from a patient with GBS during the symptomatic GBS and clinical recovery phases and subjected to single-cell RNA sequencing (scRNA-seq). The overall study design and sampling scheme are shown in **Figure 1A**, with clinical characteristics described in the Methods section. After preprocessing and quality control, 27,490 cells were retained for downstream analysis.

Unsupervised clustering identified 16 distinct cell clusters, which were visualized by uniform manifold approximation and projection (UMAP) (**Figure 1B**). Cell identities were assigned based on canonical marker gene expression (**Figure 1C**), revealing major circulating immune populations, including naïve T cells, putative double-negative T cells (DNTs), CD8^+^ T cells, cytotoxic CD8^+^ T cells, MAIT cells, CD4^+^ T cells, regulatory T cells, γδ T cells, natural killer (NK) cells, B cells, plasma cells (PCs), monocytes, megakaryocytes, and a small population of unclassified cells.

Comparison of cell-type proportions between the GBS and recovery phases revealed selective shifts in circulating immune cell composition (**Figure 1D**). Monocytes were proportionally enriched during the GBS phase relative to recovery, consistent with previous reports implicating monocytes in GBS pathogenesis (10, 19). In contrast, DNTs, CD8^+^ T cells, and NK cells represented higher relative proportions in the recovery sample than in the GBS sample, whereas naïve T cells and CD4^+^ T cells represented lower relative proportions. To evaluate intercellular signaling dynamics, we inferred cytokine-mediated cell–cell communication networks. Descriptive analysis of the inferred communication networks showed comparatively higher outgoing signaling from DNTs and PCs in the GBS-phase sample (**Figure 1E**). Monocytes showed prominent bidirectional signaling activity across both phases (**Figure 1F**). Overall, inferred outgoing cytokine signaling from PCs, monocytes, and DNTs was lower in the recovery sample.

Cell-type-specific differential expression analysis identified PCs as the population with the highest number of differentially expressed genes (DEGs), followed by monocytes (**Figure 1G**). Together, these findings reveal coordinated stage-dependent immune remodeling in PBMCs from GBS and identify plasma cells, monocytes, and DNTs as populations showing prominent transcriptional and inferred signaling differences between the paired samples.

### Result 2. Stage-dependent humoral remodeling links plasma cell activation with memory- associated B cell features in GBS

Given the central role of humoral immunity in GBS (4, 7), we examined plasma cell (PC)-specific transcriptional programs across the GBS and recovery phases.

**Figure 2:**
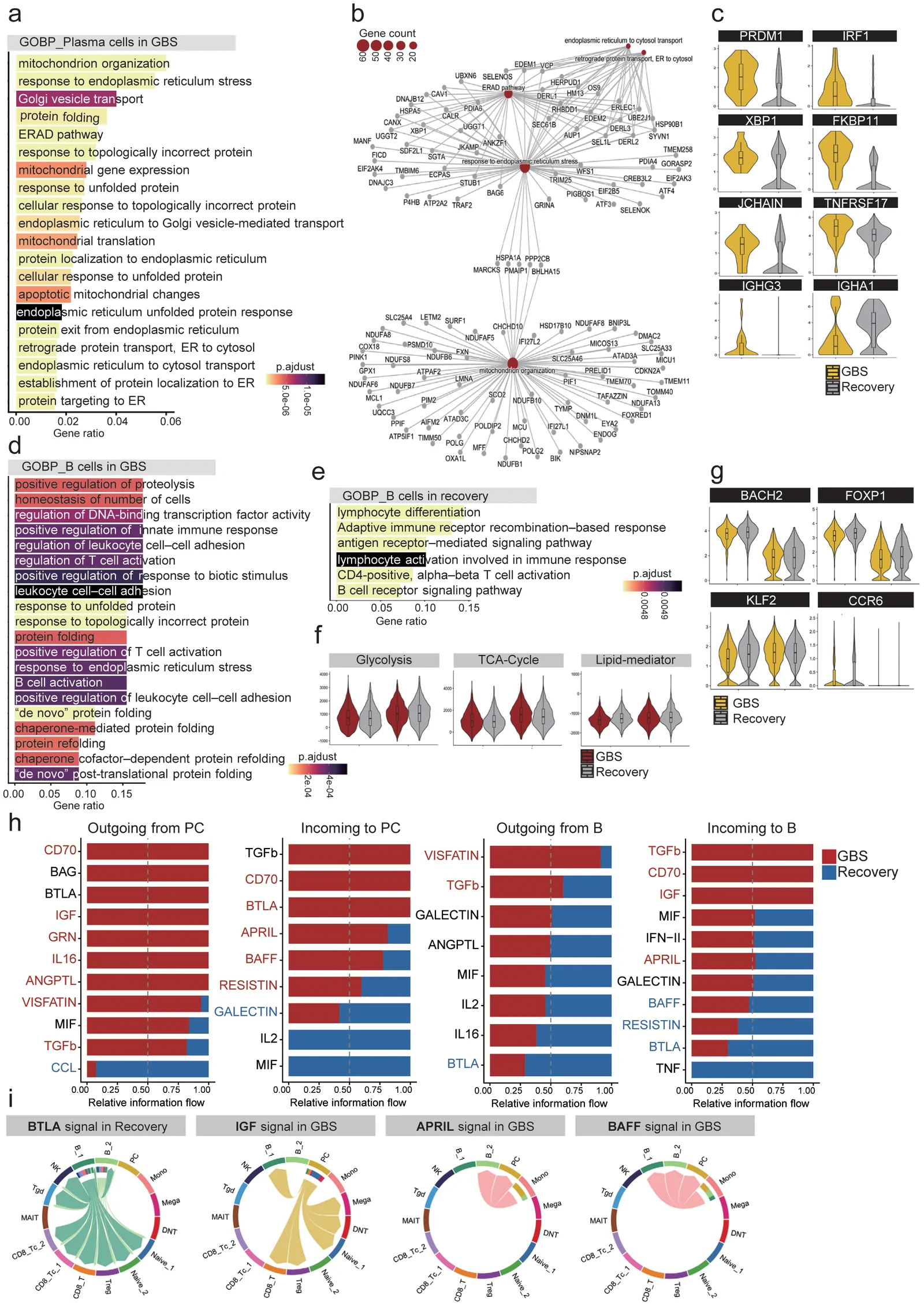
Plasma-cell activation and memory-associated B-cell transcriptional features characterize stage-associated humoral remodeling in GBS. **(A)** Bar plots showing enriched Gene Ontology Biological Process (GOBP) pathways in plasma cells during the GBS phase compared with the recovery phase (adjusted P < 0.05). **(B)** Gene–pathway network illustrating enriched functional modules in plasma cells. Red nodes represent significantly enriched biological pathways, and gray nodes represent associated genes. Edges indicate gene membership within each GO term, and node size reflects gene set size. **(C)** Violin plots showing representative plasma cell marker genes upregulated during the GBS phase. **(D)** Bar plots showing enriched GOBP pathways in B cells during the GBS phase compared with the recovery phase (adjusted P < 0.05). **(E)** Bar plots showing enriched GOBP pathways in B cells during the recovery phase compared with the GBS phase (adjusted P < 0.05). **(F)** Violin plots illustrating metabolic gene signature scores in GBS and recovery phases. **(G)** Violin plots depicting expression levels of selected immune regulatory and memory-associated genes across disease stages. **(H)** Bar plots showing enriched outgoing and incoming cytokine-mediated signaling pathways in plasma cells and B cells across GBS and recovery phases. **(I)** Circle plots illustrating cytokine-mediated intercellular communication involving plasma cells and B cells. Arrows indicate direction of signaling from sender to receiver cells, and edge thickness represents interaction strength. For panels h and i, CellChat comparisons represent descriptive within-patient observations, and no patient-level inferential statistical test was performed. For **(C, F, G)**, P values, where shown, were obtained from exploratory cell-level comparisons, and individual cells were not considered independent biological replicates.

Pathway enrichment analysis of differentially expressed genes (DEGs) revealed significant enrichment of mitochondrial organization and endoplasmic reticulum (ER)-associated protein-handling processes, including the response to unfolded proteins and ER-associated degradation (ERAD), in GBS-phase PCs (**Figures 2A, B**). Gene–pathway network analysis showed that these pathways converged on ER-associated secretory programs and mitochondrial-related modules.

Consistent with these pathway-level findings, GBS-phase PCs exhibited increased expression of plasma cell identity and secretory markers, including PRDM1, IRF4, XBP1, and FKBP11 (7, 20–23), together with elevated TNFRSF17 (BCMA) and JCHAIN, supporting enhanced antibody-secreting cell features (24, 25). GBS-phase PCs also showed an IgG-associated transcriptional profile, reflected by elevated IGHG3 expression (26), whereas recovery-phase PCs showed increased IGHA1 expression, suggesting a shift in isotype-associated transcriptional patterns (**Figure 2C**) (27). Together, these results indicate that PCs display enhanced secretory-associated transcriptional programs during the GBS phase, followed by attenuation and altered isotype-associated features during recovery.

We next examined B cells as an upstream compartment linked to plasma cell differentiation. GBS-phase B cells were enriched for protein folding and chaperone-related programs, including responses to unfolded proteins and *de novo* protein folding (**Figure 2D**). Pathways associated with B cell activation and regulation of leukocyte cell–cell adhesion were also enriched. In contrast, recovery-phase B cells showed enrichment of canonical B cell receptor (BCR) signaling pathways (**Figure 2E**).

Given that B cell activation and differentiation are accompanied by metabolic rewiring, we next compared metabolic pathway activity between the GBS and recovery phases. TCA cycle-related programs were enriched during the GBS phase, whereas lipid mediator-related pathways were enriched during recovery (**Figure 2F**) (28). Recovery-phase B cells also showed increased expression of genes associated with memory B cell transcriptional programs, including BACH2, FOXP1, KLF2, and CCR6 (29–34), with relatively higher expression in the B_1 cluster (**Figure 2G**). These transcriptional features were accompanied by an increased proportion of the B_1 cluster during recovery (**Figure 1D**).

We next examined cytokine-mediated cell–cell interactions involving PCs and B cells (**Figures 2H, I**). PCs showed increased insulin-like growth factor (IGF) signaling activity during the GBS phase (35). BTLA-mediated inhibitory signaling (36, 37) was detectable in both phases but became more broadly distributed across immune populations during recovery, particularly among B cells. In the recalculated CellChat analysis performed without population-size weighting, inferred incoming BAFF- and APRIL-associated signaling toward B cells and PCs remained comparatively higher in the GBS-phase sample, with monocytes identified as a candidate source. These results represent descriptive within-patient observations. Consistent with this pattern, BAFF and APRIL are established regulators of B cell survival and differentiation (35, 38–40) and are primarily produced by antigen-presenting cells, including monocytes (41).

Taken together, these findings describe distinct immune features observed across the paired samples from this patient, including ER-associated plasma cell activation and inferred monocyte-derived BAFF/APRIL signaling during the GBS phase, followed by the emergence of memory-associated B cell features and broader inhibitory signaling during recovery.

### Result 3. Monocytes form a stage-dependent myeloid axis linking inflammatory activation and humoral signaling in GBS

Given prior reports implicating monocytes in GBS pathogenesis (42) and the monocyte expansion and myeloid activation signatures observed in our dataset (**Figures 1**, **2**), we examined monocyte-specific transcriptional programs across the GBS and recovery phases.

**Figure 3:**
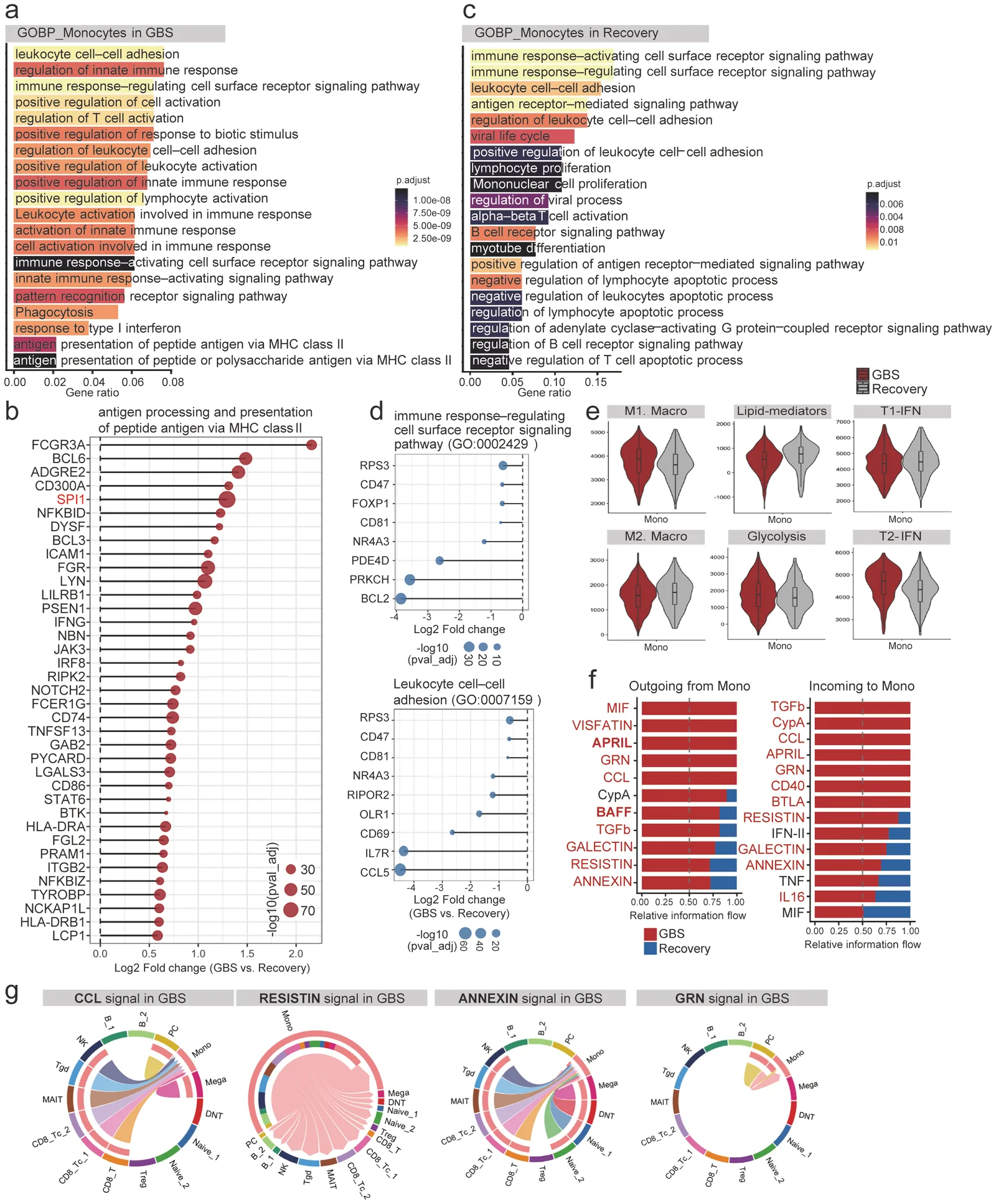
Stage-dependent transcriptional reprogramming of monocytes in GBS. **(A)** Bar plots showing enriched Gene Ontology Biological Process (GOBP) pathways in monocytes during the GBS phase compared with recovery (adjusted P < 0.05). **(B)** Lollipop plot illustrating upregulated functional modules in monocytes in the GBS sample, including antigen processing and presentation of peptide antigen via MHC class II. **(C)** Bar plots showing enriched GOBP pathways in monocytes during the recovery phase compared with the GBS phase (adjusted P < 0.05). **(D)** Lollipop plots illustrating enriched biological processes in recovery-phase monocytes, including immune response–regulating cell surface receptor signaling and leukocyte cell–cell adhesion. **(E)** Violin plots showing representative gene expression differences between GBS and recovery phases in monocytes. **(F)** Bar plots depicting enriched outgoing and incoming cytokine-mediated signaling pathways in monocytes across disease stages. **(G)** Circle plots illustrating cytokine-mediated communication networks highlighting monocytes during GBS and recovery phases. Arrows indicate directionality of signaling, and edge thickness represents interaction strength. For **(F, G)**, CellChat comparisons represent descriptive within-patient observations, and no patient-level inferential statistical test was performed. Gene-expression comparisons presented in **(B, E)** were exploratory cell-level analyses, and individual cells were not considered independent biological replicates.

During the GBS phase, monocytes showed significant enrichment of pro-inflammatory pathways related to innate immune activation, antigen processing and presentation, and immune cell activation compared with the recovery phase (**Figure 3A**).

Gene-level analysis identified upregulation of canonical antigen-presenting genes, including HLA-DRA, a key component of MHC class II-mediated antigen presentation (43), together with increased SPI1 expression (**Figure 3B**). SPI1 (PU.1) is a lineage-defining transcription factor essential for myeloid differentiation and inflammatory gene regulation (44), consistent with an activated myeloid phenotype in GBS-phase monocytes. TNFSF13, which encodes APRIL, was also upregulated (**Figure 3B**), linking GBS-phase monocytes to potential humoral immune regulation.

In contrast, recovery-phase monocytes showed reduced antigen-presentation programs and increased expression of regulatory- and survival-associated genes, including FOXP1 and BCL2 (45, 46) (**Figures 3C, D**).

To further characterize activation and metabolic states, we used the ESCAPE database to quantify macrophage-related transcriptional signatures (**Figure 3E**). GBS-phase monocytes exhibited higher scores for the M1-associated macrophage transcriptional signature (M1.Macro), glycolysis, and type II interferon (T2-IFN) signatures, consistent with inflammatory myeloid activation and metabolic reprogramming toward glycolysis (28, 47). During recovery, monocytes showed higher scores for the M2-associated macrophage transcriptional signature (M2.Macro) and lipid metabolism-related signatures, features commonly associated with resolution-phase or regulatory myeloid states (28, 47).

We next examined cytokine-mediated cell–cell interactions to characterize monocyte-centered communication dynamics. During the GBS phase, monocytes displayed enhanced cytokine and chemokine signaling involving CCL family pathways (**Figures 3F, G**) which are known mediators of leukocyte recruitment and inflammatory amplification (48). Consistent with the findings presented in **Figure 2**, inferred monocyte-derived BAFF and APRIL signaling toward B cells and plasma cells was prominently enriched during the GBS phase, further linking monocytes to the humoral activation network (**Figure 3F**).

In addition, RESISTIN, ANNEXIN, and GRN signaling pathways were enriched in both the GBS and recovery phases, particularly in outgoing interactions from monocytes (**Figure 3G**, **Supplementary Figure 1B**). These mediators have been reported to participate in context-dependent immunomodulatory signaling networks and to increase during monocyte-to-macrophage differentiation (49–53), suggesting sustained monocyte involvement in immune regulation beyond GBS inflammatory activation.

Together, these findings indicate that monocytes undergo stage-dependent transcriptional reprogramming in GBS, shifting from an inflammatory, antigen-presenting phenotype during the GBS phase toward a regulatory-associated state during recovery. Beyond classical inflammatory activation, GBS-phase monocytes also displayed inferred humoral-supporting signals, including BAFF/APRIL-associated communication toward B cells and plasma cells. These findings nominate monocytes as a stage-dependent myeloid axis associated with inflammatory activation and humoral immune remodeling in GBS.

### Result 4. SPI1-associated regulatory network enrichment in inflammatory monocytes during the GBS phase

**Figure 4:**
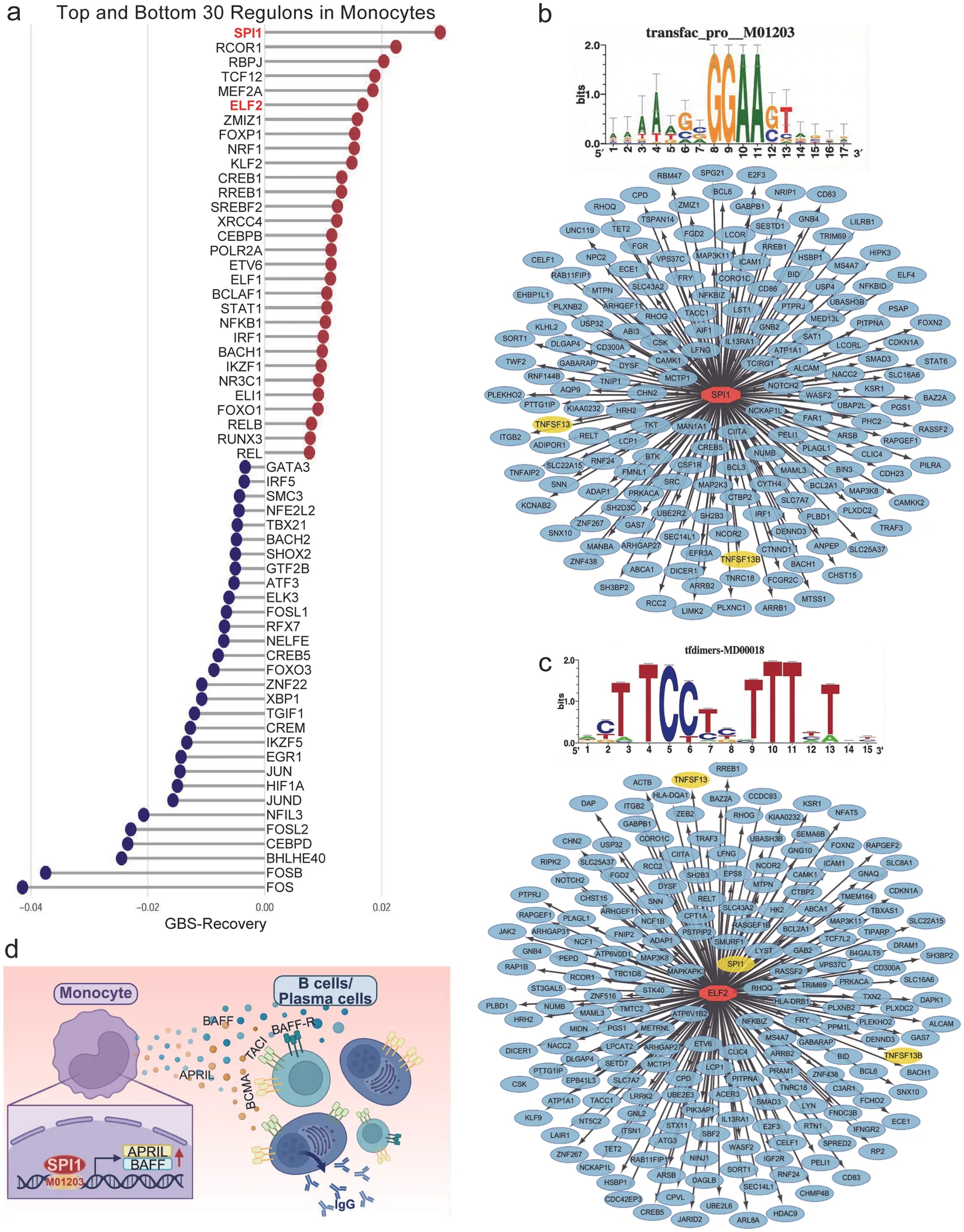
SPI1-associated regulatory network enrichment in inflammatory monocytes during GBS. **(A)** Lollipop plot showing regulon activity scores in monocytes, highlighting transcription factors with significantly increased or decreased activity during the GBS phase. **(B)** Sequence logo plot representing the most enriched transcription factor binding motif identified in monocytes in the GBS sample. Network map illustrates predicted SPI1 target genes inferred from regulon analysis. Nodes represent SPI1 and its target genes, and edges indicate regulatory relationships. TNFSF13 (APRIL) and TNFSF13B (BAFF) are highlighted in yellow. **(C)** Sequence logo plot showing the second most enriched motif in disease-phase monocytes. **(D)** Schematic illustration of the proposed SPI1-associated monocyte–B-cell regulatory axis involving BAFF/APRIL signaling during the GBS phase. The corresponding network map illustrates predicted target genes regulated by ELF2 and SPI1. TNFSF13 and TNFSF13B are highlighted in yellow. Regulon activity comparisons in panel a represent exploratory cell-level analyses, and individual cells were not considered independent biological replicates. The motif and target-gene relationships shown in panels b and c are computational predictions and do not establish direct transcriptional regulation.

To define monocyte-specific gene regulatory networks, we performed single-cell regulatory network inference and clustering (SCENIC) analysis (16). Among the regulons enriched in GBS-phase monocytes, SPI1 emerged as one of the most prominent transcription factors (**Figure 4A**).

To further assess transcription factor–motif associations, we applied the iRegulon package in Cytoscape using differentially expressed genes from GBS-phase monocytes (**Figure 4B**) (17, 18). Motif enrichment analysis identified the TRANSFAC Pro motif M01203 as significantly enriched among GBS-phase monocyte DEGs, with SPI1 predicted as the top associated transcription factor. Notably, TNFSF13 (APRIL) and TNFSF13B (BAFF) were among the predicted target genes linked to this motif.

In addition, analysis of ligand-associated sequence motifs revealed enrichment of the MD00018 motif from the TFDIMERS database, which was associated with TNFSF13, TNFSF13B, and SPI1 (**Figure 4C**). These findings suggest that SPI1-associated regulatory motifs are enriched in GBS-phase monocytes and may be linked to transcriptional networks involving BAFF/APRIL-related genes.

Collectively, these findings identify SPI1-associated regulatory activity within inflammatory monocyte programs observed in the GBS sample and suggest a potential association between myeloid activation and BAFF/APRIL-related humoral signaling (**Figure 4D**).

### Result 5. Stage-associated transcriptional remodeling of putative double-negative T cells across the GBS and recovery phases

Although putative double-negative T (DNT) cells represent a relatively small fraction of human PBMCs, they have been reported to exhibit context-dependent inflammatory and regulatory features in autoimmune settings (54–56). To characterize their transcriptional dynamics in GBS, we performed Gene Ontology Biological Process (GOBP) analysis across the GBS and recovery phases.

**Figure 5:**
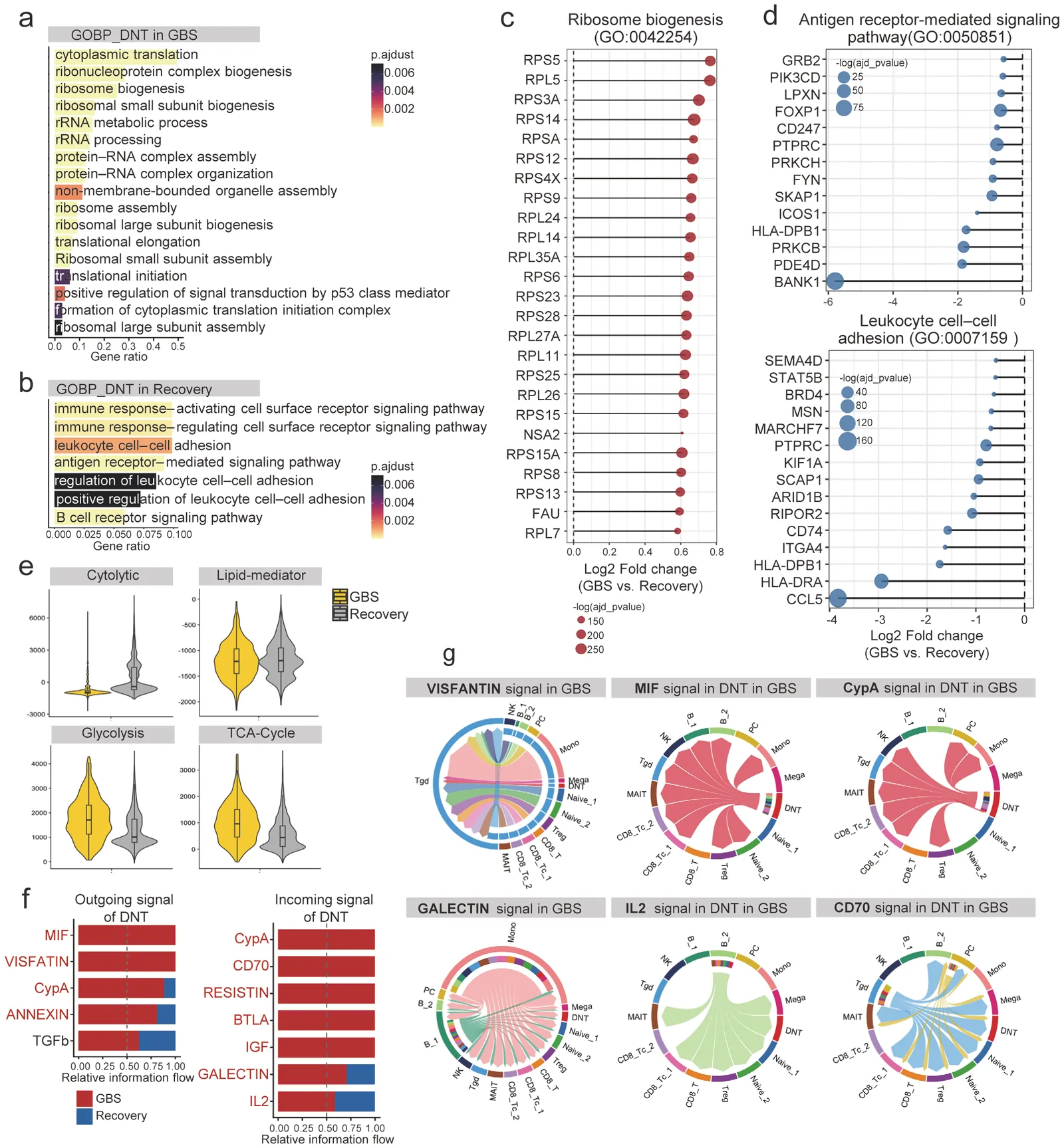
Putative double-negative T cells show stage-associated transcriptional and inferred signaling differences across the GBS and recovery phases. **(A)** Bar plots showing enriched Gene Ontology Biological Process (GOBP) pathways in DNT cells during the GBS phase compared with recovery (adjusted P < 0.05). **(B)** Bar plots showing enriched GOBP pathways in DNT cells during the recovery phase compared with the GBS phase (adjusted P < 0.05). **(C)** Lollipop plot illustrating upregulated pathways in DNT cells in the GBS sample, including ribosome biogenesis and associated genes. **(D)** Lollipop plot illustrating upregulated pathways in recovery-phase DNT cells, including antigen receptor–mediated signaling and leukocyte cell–cell adhesion, together with associated genes. **(E)** Violin plots showing metabolic gene signature scores in DNT cells across disease stages. **(F)** Bar charts displaying the top five outgoing and incoming cytokine-mediated signaling pathways in DNT cells. **(G)** Circle plots illustrating cytokine-mediated intercellular communication networks across immune populations. Arrows indicate directionality of signaling from sender to receiver cells, and edge thickness represents interaction strength. For **(F, G)**, CellChat comparisons represent descriptive within-patient observations, and no patient-level inferential statistical test was performed. For panel **(E)**, P values, where shown, were obtained from exploratory cell-level comparisons, and individual cells were not considered independent biological replicates.

During the GBS phase, DNTs showed significant enrichment of protein synthesis-related pathways, including cytoplasmic translation and ribosome biogenesis (**Figures 5A, C**), consistent with an activation-associated transcriptional signature during the GBS phase. In contrast, recovery-phase DNTs exhibited enrichment of immune receptor-mediated signaling and leukocyte cell–cell adhesion pathways (**Figures 5B, D**), transcriptional features associated with contact-dependent immune interactions (57, 58).

Metabolic and functional signature analysis demonstrated higher biosynthetic, glycolysis, and tricarboxylic acid (TCA) cycle-related activity scores during the GBS phase (**Figure 5E**). During recovery, cytolytic activity scores were increased together with effector-associated transcriptional programs. Lipid-mediated metabolic signatures were also relatively elevated during recovery, suggesting a stage-dependent metabolic shift (28).

In parallel with these transcriptional changes, DNTs represented a higher relative proportion in the recovery sample than in the GBS sample (**Figure 1D**), consistent with a difference in immune-cell composition between the two samples.

Consistent with earlier observations (**Figures 1E, F**), DNTs in the GBS sample showed comparatively higher inferred outgoing cytokine-mediated signaling to other immune populations, including MIF, VISFATIN, and Cyclophilin A (CypA) pathways (**Figures 5F, G**) (59–61). DNTs in the GBS sample also showed stronger inferred incoming ANNEXIN and RESISTIN signaling (51, 62), indicating broader intercellular communication involving DNTs during the GBS phase.

Regulatory and immunomodulatory pathways, including BTLA and TGF-β (36, 37, 63), as well as IGF- and IL-2–related signaling (64, 65), were detected in both outgoing and incoming communication networks, indicating the coexistence of inflammatory and regulatory signaling components. In contrast, other T cell subsets and NK cells displayed increased cytokine-mediated interactions and activation-associated signaling during the GBS phase compared with recovery (**Supplementary Figures 2**-**4**), consistent with inflammatory activation patterns observed in GBS.

Together, these findings suggest that DNTs exhibited stage-associated shifts in transcriptional programs, with GBS phase signatures enriched for cytokine-related signaling and recovery phase signatures associated with receptor-mediated signaling, cell adhesion, and effector functions. These patterns indicate a potential change in the transcriptional state of DNTs across disease stages.

## Discussion

Guillain–Barré syndrome (GBS) is characterized by pathogenic autoantibody production and systemic immune dysregulation. However, how peripheral immune populations change in a coordinated manner across the GBS and recovery phases remains incompletely defined. In this longitudinal single-cell transcriptomic analysis of PBMCs from a patient with GBS, we observed coordinated remodeling across humoral, myeloid, and lymphoid compartments. The GBS phase was associated with enhanced plasma cell activity and inflammatory monocyte features, whereas the recovery sample showed partial attenuation of these programs and a shift toward more regulated immune states.

Plasma cells (PCs), as antibody-secreting effectors, are implicated in the humoral component of GBS pathogenesis. In our dataset, PCs in the GBS phase showed higher expression of secretory pathway components and IgG-associated genes, consistent with enhanced antibody-secreting activity. These findings support stage-associated PC modulation, with stronger secretory-associated programs in the GBS phase and attenuation of these programs during recovery.

B cells displayed transcriptional features consistent with metabolic and biosynthetic priming during the GBS phase, whereas canonical B cell receptor (BCR) signaling and memory-associated programs were enriched during recovery. In parallel, inferred BTLA-mediated inhibitory signaling was more broadly represented during recovery, particularly within the B_1 cluster, which exhibited memory-associated transcriptional features. BTLA signaling in memory B cells has been associated with suppression of adaptive immune activation in autoimmune contexts (66). Its enrichment in recovery-phase B cells may reflect a transition toward a more regulated humoral immune state.

During the GBS phase, the immune profile was characterized by comparatively higher inferred BAFF/APRIL-mediated intercellular communication. The BAFF/APRIL axis regulates B cell survival and differentiation through BAFF-R, TACI, and BCMA; BAFF-R promotes survival via alternative NF-κB signaling, whereas BCMA supports terminal differentiation and maintenance of long-lived plasma cells (38, 39). Thus, the comparatively higher inferred BAFF/APRIL signaling observed during the GBS phase may be associated with antibody-secreting activity. Given that monocytes are a major source of these ligands (67), the comparatively higher inferred outgoing BAFF/APRIL signaling observed in our dataset suggests a potential monocyte-derived input into humoral activation.

Monocytes in the GBS phase showed enrichment of antigen-presentation pathways, higher TNFSF13 (APRIL) expression, and greater similarity to an M1-associated transcriptional signature coupled with enhanced glycolysis, consistent with inflammatory myeloid activation (47). Gene regulatory network analysis nominated SPI1 (PU.1) as a candidate transcription factor associated with this phenotype. The association of SPI1 activity with motifs linked to TNFSF13 and TNFSF13B further suggests a potential relationship between inflammatory myeloid transcriptional state and BAFF/APRIL-related humoral signaling.

During the recovery phase, monocytes displayed reduced inflammatory signatures together with enrichment of regulatory-associated pathways, higher scores for an M2-associated transcriptional signature, and metabolic features consistent with anti-inflammatory or resolution-associated states (47). Intercellular communication analysis further indicated their participation in bidirectional signaling networks, including modulatory pathways such as ANNEXIN- and RESISTIN-mediated signaling. These findings suggest that circulating monocytes do not represent a uniformly inflammatory population during the GBS phase, but instead exhibit stage-associated differences across inflammatory and regulatory-associated states. Together, our longitudinal profiles highlight monocytes as a prominent component of the observed immune changes and suggest a potential link between myeloid activation and humoral-supporting communication through SPI1-associated regulatory programs.

The putative double-negative T-cell (DNT) population also showed stage-associated transcriptional differences. Although DNTs represent a minor peripheral T-cell population and have been linked to pro-inflammatory expansion in autoimmune disease (55), DNTs represented a higher relative proportion in the recovery sample than in the GBS sample. DNTs in the recovery sample were enriched for cell–cell adhesion-related genes and displayed metabolic features consistent with recovery-associated immune reorganization, suggesting context-dependent immunomodulatory transcriptional features.

In contrast, DNTs in the GBS sample showed higher expression of inflammatory mediators, including MIF, a ligand implicated in B-cell survival (68), whereas DNTs in the recovery sample showed less prominent activation-associated transcriptional features. These observations describe stage-associated differences in the transcriptional signatures and inferred signaling patterns of the putative DNT population.

**Figure 6:**
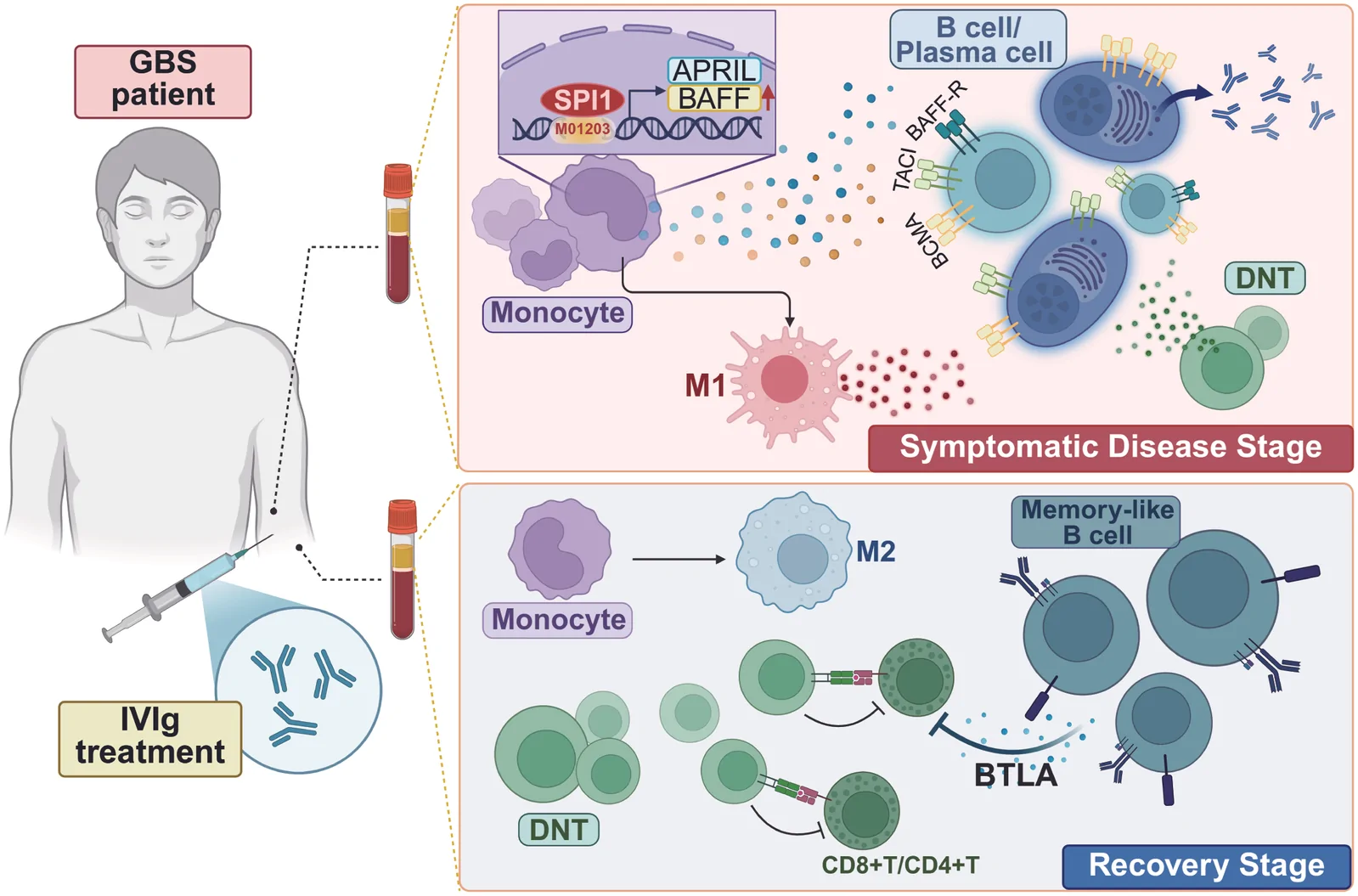
Graphical summary of longitudinal immune alterations across the GBS and recovery phases. PBMCs collected during the symptomatic GBS and clinical recovery phases were analyzed using single-cell RNA sequencing. The analysis indicated inflammatory monocyte activation and BAFF/APRIL-associated immune-cell communication during the GBS phase. During recovery, the immune landscape showed more regulatory-associated transcriptional features, together with changes in monocyte transcriptional-state signatures, B-cell composition, and lymphocyte interactions. The schematic summarizes the major cell states and interactions across two phases.

Our findings partly recapitulate previously reported myeloid activation in GBS but extend these observations by demonstrating that this myeloid state is embedded within broader longitudinal remodeling of humoral and lymphoid immune compartments. Paired analysis across two clinical time points revealed coordinated changes in plasma cell secretory programs, monocyte-derived BAFF/APRIL communication with B cells and plasma cells, SPI1-associated myeloid regulation, and DNT-associated inferred cytokine signaling. Thus, the contribution of this study lies not in identifying monocyte activation per se, but in delineating the temporal reorganization of a coordinated immune network involving monocytes, B cells and plasma cells, and putative DNTs (**Figure 6**).

Current therapies for GBS, including intravenous immunoglobulin (IVIg) and plasma exchange (PE), are primarily thought to neutralize or remove circulating pathogenic factors. However, how these interventions reshape cellular immune programs remains incompletely understood. In this context, our analysis provides a cellular-level view of the changes observed between the GBS and recovery phases and highlights altered monocyte-B communication as one of the immune features that may merit further investigation.

The rarity of GBS and the difficulty of obtaining paired longitudinal samples limited this study to a single-patient design. Because this study included only one patient sampled at two time points, individual cells did not constitute independent biological replicates. Accordingly, cell-level differential-expression statistics and CellChat comparisons should be interpreted as exploratory and descriptive within-patient observations rather than evidence of biological reproducibility. Differences in cell abundance and composition may also affect the stability of inferred communication networks. In addition, because the two samples were collected at distinct clinical time points without matched controls, the observed differences cannot be disentangled from the effects of IVIg exposure, natural recovery, resolution of the preceding illness, ICU-related factors, mechanical ventilation, or technical variation between samples. Additional biospecimens were not available for orthogonal protein-level or flow-cytometric validation. Therefore, the inferred BAFF/APRIL-associated communication, SPI1 regulatory activity, and DNT-associated signaling patterns should be regarded as hypothesis-generating computational predictions requiring confirmation in independent samples. Although this analysis provides a stage-resolved view of peripheral immune dynamics, the broader applicability of the observed signatures should be interpreted in the context of the clinical and immunological heterogeneity of GBS. Nevertheless, the paired longitudinal design enabled the characterization of coordinated immune changes within the same patient while reducing the influence of interindividual variability. Validation in larger independent cohorts and functional studies will be necessary to determine the generalizability and mechanistic relevance of these findings.

## Data Availability

The datasets presented in this study can be found in online repositories. The names of the repository/repositories and accession number(s) can be found below: https://www.ncbi.nlm.nih.gov/, GSE334853.
