# Supplementary material for: Monocyte–plasma cell communication characterizes treatment-associated peripheral immune remodeling in Guillain–Barré syndrome
Source: Front Immunol. 2026 Aug 13;17:1931955. doi: 10.3389/fimmu.2026.1931955 (PMC13518544; doi:10.3389/fimmu.2026.1931955)
Supplement: Supplementary file 1 [file DataSheet1.pdf]

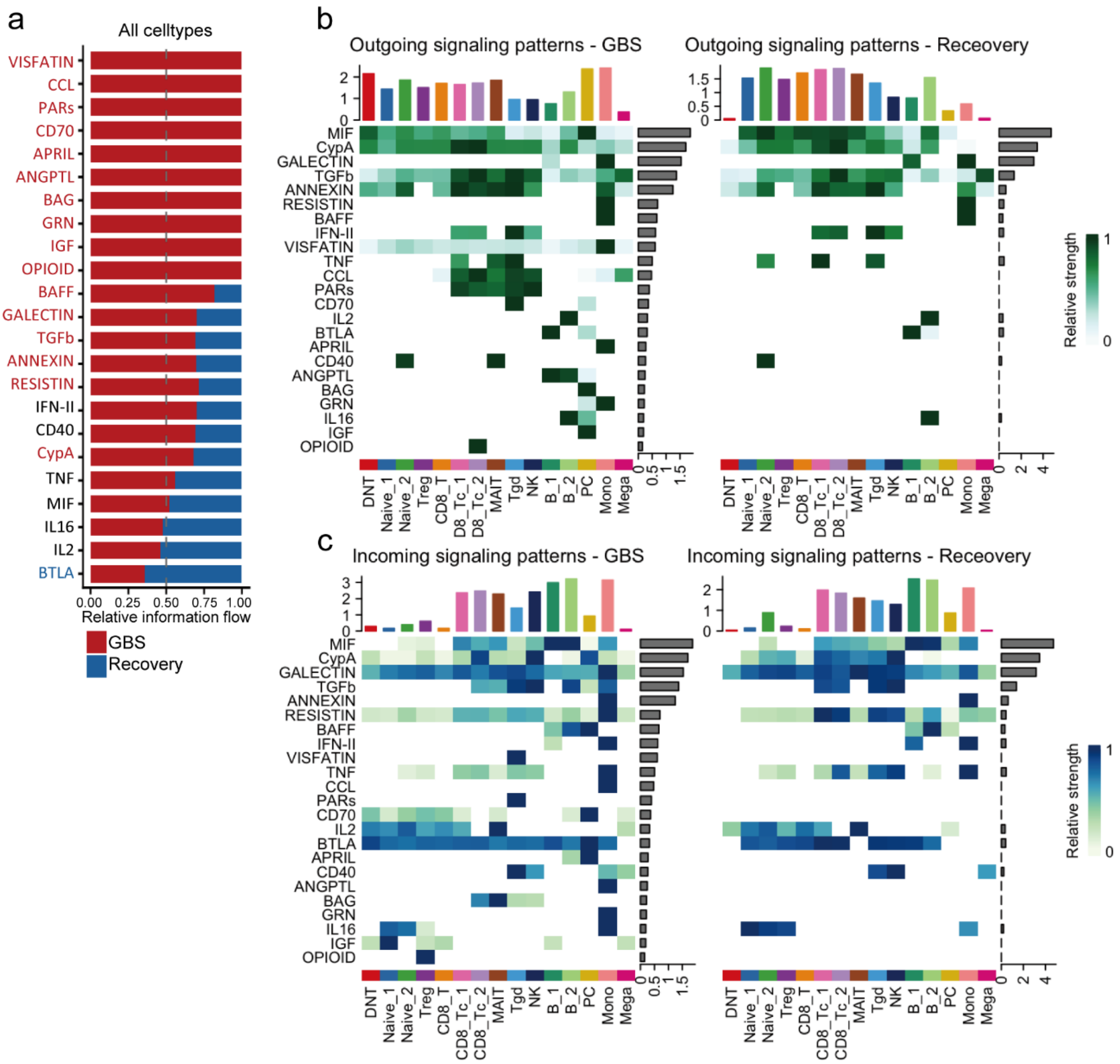

Supplementary Figure S1. Global cytokine-mediated communication patterns across the GBS and recovery samples.

a. Bar plot showing relative outgoing and incoming cytokine-mediated signaling pathway information flow across all cell types in the GBS and recovery samples. **b.** Heatmaps illustrating outgoing cytokine-mediated signaling patterns across annotated cell populations in the GBS and recovery samples. Color intensity represents relative signaling strength. **c.** Heatmaps illustrating incoming cytokine-mediated signaling patterns across annotated cell populations in the GBS and recovery samples. Color intensity represents relative signaling strength.

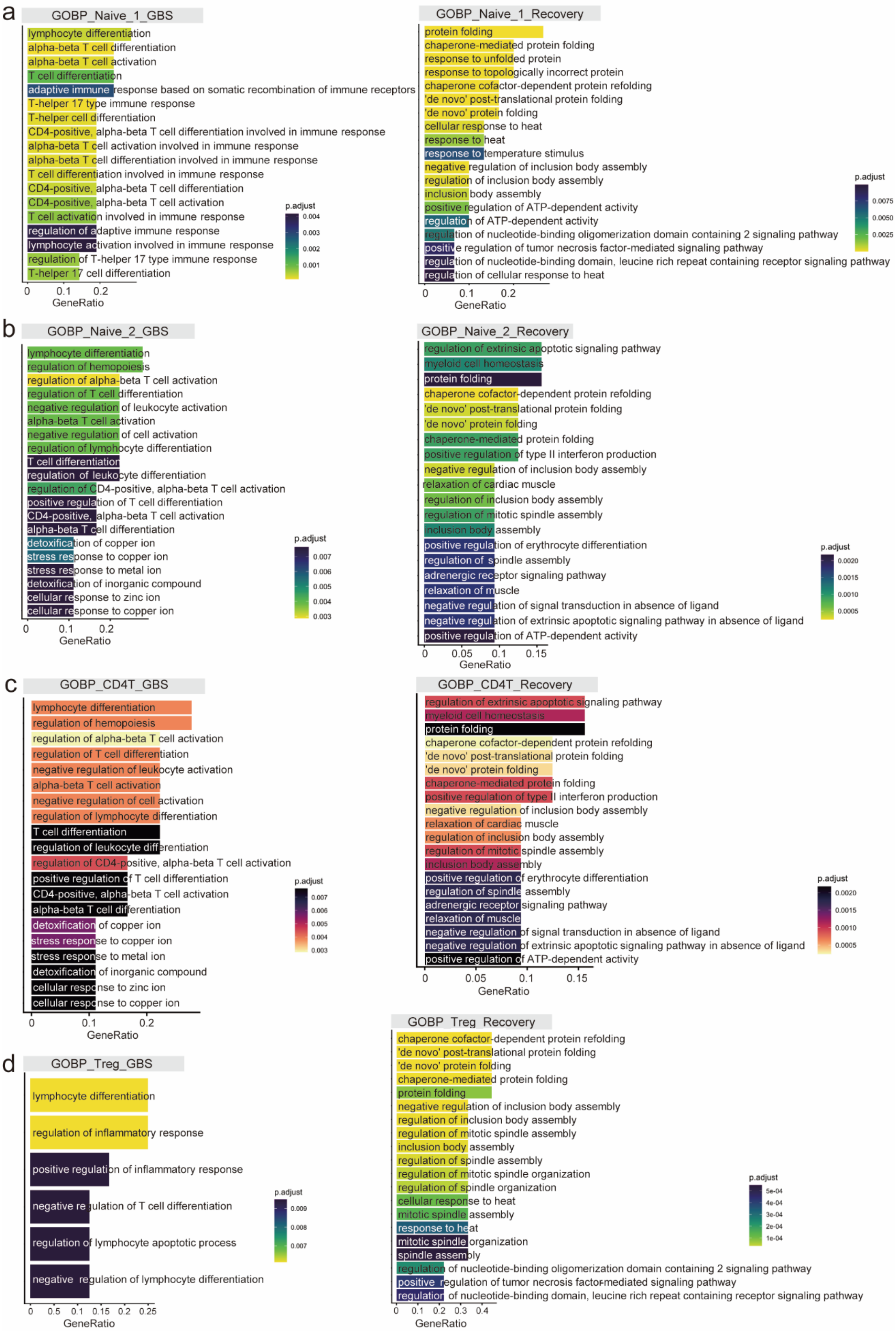
a
GOBP_Naive_1_GBS
lymphocyte differentiation
alpha-beta T cell differentiation
alpha-beta T cell activation
T cell differentiation
adaptive immune response based on somatic recombination of immune receptors
T-helper 17 type immune response
T-helper cell differentiation
CD4-positive, alpha-beta T cell differentiation involved in immune response
alpha-beta T cell activation involved in immune response
alpha-beta T cell differentiation involved in immune response
T cell differentiation involved in immune response
CD4-positive, alpha-beta T cell differentiation
CD4-positive, alpha-beta T cell activation
T cell activation involved in immune response
regulation of adaptive immune response
lymphocyte activation involved in immune response
regulation of T-helper 17 type immune response
T-helper 17 cell differentiation
p.adjust
0.004
0.003
0.002
0.001
0
0.1
0.2
GeneRatio
GOBP_Naive_1_Recovery
protein folding
chaperone-mediated protein folding
response to unfolded protein
response to topologically incorrect protein
chaperone cofactor-dependent protein refolding
'de novo' post-translational protein folding
'de novo' protein folding
cellular response to heat
response to heat
response to temperature stimulus
negative regulation of inclusion body assembly
regulation of inclusion body assembly
inclusion body assembly
positive regulation of ATP-dependent activity
regulation of ATP-dependent activity
regulation of nucleotide-binding oligomerization domain containing 2 signaling pathway
positive regulation of tumor necrosis factor-mediated signaling pathway
regulation of nucleotide-binding domain, leucine rich repeat containing receptor signaling pathway
regulation of cellular response to heat
p.adjust
0.0075
0.0050
0.0025
0
0.1
0.2
GeneRatio
b
GOBP_Naive_2_GBS
lymphocyte differentiation
regulation of hemopoiesis
regulation of alpha-beta T cell activation
regulation of T cell differentiation
negative regulation of leukocyte activation
alpha-beta T cell activation
negative regulation of cell activation
regulation of lymphocyte differentiation
T cell differentiation
regulation of leukocyte differentiation
regulation of CD4-positive, alpha-beta T cell activation
positive regulation of T cell differentiation
CD4-positive, alpha-beta T cell activation
alpha-beta T cell differentiation
detoxification of copper ion
stress response to copper ion
stress response to metal ion
detoxification of inorganic compound
cellular response to zinc ion
cellular response to copper ion
p.adjust
0.007
0.006
0.005
0.004
0.003
0
0.1
0.2
GeneRatio
GOBP_Naive_2_Recovery
regulation of extrinsic apoptotic signaling pathway
myeloid cell homeostasis
protein folding
chaperone cofactor-dependent protein refolding
'de novo' post-translational protein folding
'de novo' protein folding
chaperone-mediated protein folding
positive regulation of type II interferon production
negative regulation of inclusion body assembly
relaxation of cardiac muscle
regulation of inclusion body assembly
regulation of mitotic spindle assembly
inclusion body assembly
positive regulation of erythrocyte differentiation
regulation of spindle assembly
adrenergic receptor signaling pathway
relaxation of muscle
negative regulation of signal transduction in absence of ligand
negative regulation of extrinsic apoptotic signaling pathway in absence of ligand
positive regulation of ATP-dependent activity
p.adjust
0.0020
0.0015
0.0010
0.0005
0
0.05
0.1
0.15
GeneRatio
c
GOBP_CD4T_GBS
lymphocyte differentiation
regulation of hemopoiesis
regulation of alpha-beta T cell activation
regulation of T cell differentiation
negative regulation of leukocyte activation
alpha-beta T cell activation
negative regulation of cell activation
regulation of lymphocyte differentiation
T cell differentiation
regulation of leukocyte differentiation
regulation of CD4-positive, alpha-beta T cell activation
positive regulation of T cell differentiation
CD4-positive, alpha-beta T cell activation
alpha-beta T cell differentiation
detoxification of copper ion
stress response to copper ion
stress response to metal ion
detoxification of inorganic compound
cellular response to zinc ion
cellular response to copper ion
p.adjust
0.007
0.006
0.005
0.004
0.003
0
0.1
0.2
GeneRatio
GOBP_CD4T_Recovery
regulation of extrinsic apoptotic signaling pathway
myeloid cell homeostasis
protein folding
chaperone cofactor-dependent protein refolding
'de novo' post-translational protein folding
'de novo' protein folding
chaperone-mediated protein folding
positive regulation of type II interferon production
negative regulation of inclusion body assembly
relaxation of cardiac muscle
regulation of inclusion body assembly
regulation of mitotic spindle assembly
inclusion body assembly
positive regulation of erythrocyte differentiation
regulation of spindle assembly
adrenergic receptor signaling pathway
relaxation of muscle
negative regulation of signal transduction in absence of ligand
negative regulation of extrinsic apoptotic signaling pathway in absence of ligand
positive regulation of ATP-dependent activity
p.adjust
0.0020
0.0015
0.0010
0.0005
0
0.05
0.1
0.15
GeneRatio
d
GOBP_Treg_GBS
lymphocyte differentiation
regulation of inflammatory response
positive regulation of inflammatory response
negative regulation of T cell differentiation
regulation of lymphocyte apoptotic process
negative regulation of lymphocyte differentiation
p.adjust
0.009
0.008
0.007
0
0.5
0.1
0.15
0.2
0.25
GeneRatio
GOBP_Treg_Recovery
chaperone cofactor-dependent protein refolding
'de novo' post-translational protein folding
'de novo' protein folding
chaperone-mediated protein folding
protein folding
negative regulation of inclusion body assembly
regulation of inclusion body assembly
regulation of mitotic spindle assembly
inclusion body assembly
regulation of spindle assembly
regulation of mitotic spindle organization
regulation of spindle organization
cellular response to heat
mitotic spindle assembly
response to heat
mitotic spindle organization
spindle assembly
regulation of nucleotide-binding oligomerization domain containing 2 signaling pathway
positive regulation of tumor necrosis factor-mediated signaling pathway
regulation of nucleotide-binding domain, leucine rich repeat containing receptor signaling pathway
p.adjust
5e-04
4e-04
3e-04
2e-04
1e-04
0
0.1
0.2
0.3
0.4
GeneRatio

Supplementary Figure S2. Gene Ontology enrichment analysis of naïve, CD4$^+$, and regulatory T-cell populations.

a–d. Bar plots showing enriched Gene Ontology Biological Process pathways in naïve T-cell subsets (Naïve_1 and Naïve_2), CD4$^+$ T cells, and regulatory T cells in the GBS and recovery samples. Adjusted P values indicate over-representation of Gene Ontology terms within the exploratory cell-level DEG lists. Because individual cells were not considered independent biological replicates, these enrichment results do not represent patient-level biological inference.

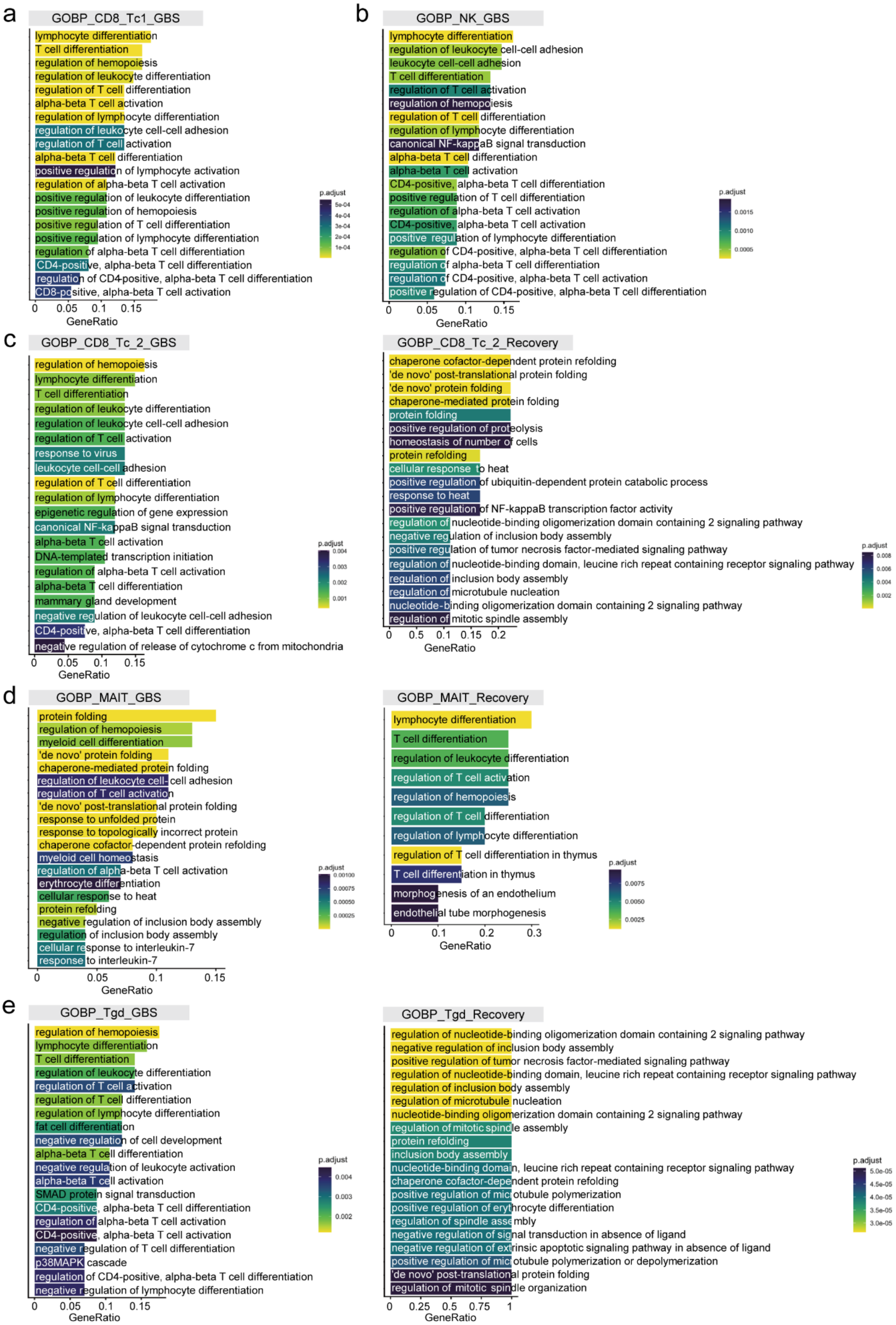

a
GOBP_CD8_Tc1_GBS
lymphocyte differentiation
T cell differentiation
regulation of hemopoiesis
regulation of leukocyte differentiation
regulation of T cell differentiation
alpha-beta T cell activation
regulation of lymphocyte differentiation
regulation of leukocyte cell-cell adhesion
regulation of T cell activation
alpha-beta T cell differentiation
positive regulation of lymphocyte activation
regulation of alpha-beta T cell activation
positive regulation of leukocyte differentiation
positive regulation of hemopoiesis
positive regulation of T cell differentiation
positive regulation of lymphocyte differentiation
regulation of alpha-beta T cell differentiation
CD4-positive, alpha-beta T cell differentiation
regulation of CD4-positive, alpha-beta T cell differentiation
CD8-positive, alpha-beta T cell activation
0 0.05 0.1 0.15
GeneRatio
p.adjust
b
GOBP_NK_GBS
lymphocyte differentiation
regulation of leukocyte cell-cell adhesion
leukocyte cell-cell adhesion
T cell differentiation
regulation of T cell activation
regulation of hemopoiesis
regulation of T cell differentiation
regulation of lymphocyte differentiation
canonical NF-kappaB signal transduction
alpha-beta T cell differentiation
alpha-beta T cell activation
CD4-positive, alpha-beta T cell differentiation
positive regulation of T cell differentiation
regulation of alpha-beta T cell activation
CD4-positive, alpha-beta T cell activation
positive regulation of lymphocyte differentiation
regulation of CD4-positive, alpha-beta T cell differentiation
regulation of alpha-beta T cell differentiation
regulation of CD4-positive, alpha-beta T cell activation
positive regulation of CD4-positive, alpha-beta T cell differentiation
0 0.05 0.1 0.15
GeneRatio
p.adjust
c
GOBP_CD8_Tc_2_GBS
regulation of hemopoiesis
lymphocyte differentiation
T cell differentiation
regulation of leukocyte differentiation
regulation of leukocyte cell-cell adhesion
regulation of T cell activation
response to virus
leukocyte cell-cell adhesion
regulation of T cell differentiation
regulation of lymphocyte differentiation
epigenetic regulation of gene expression
canonical NF-kappaB signal transduction
alpha-beta T cell activation
DNA-templated transcription initiation
regulation of alpha-beta T cell activation
alpha-beta T cell differentiation
mammary gland development
negative regulation of leukocyte cell-cell adhesion
CD4-positive, alpha-beta T cell differentiation
negative regulation of release of cytochrome c from mitochondria
0 0.05 0.1 0.15
GeneRatio
p.adjust
GOBP_CD8_Tc_2_Recovery
chaperone cofactor-dependent protein refolding
'de novo' post-translational protein folding
'de novo' protein folding
chaperone-mediated protein folding
protein folding
positive regulation of proteolysis
homeostasis of number of cells
protein refolding
cellular response to heat
positive regulation of ubiquitin-dependent protein catabolic process
response to heat
positive regulation of NF-kappaB transcription factor activity
regulation of nucleotide-binding oligomerization domain containing 2 signaling pathway
negative regulation of inclusion body assembly
positive regulation of tumor necrosis factor-mediated signaling pathway
regulation of nucleotide-binding domain, leucine rich repeat containing receptor signaling pathway
regulation of inclusion body assembly
regulation of microtubule nucleation
nucleotide-binding oligomerization domain containing 2 signaling pathway
regulation of mitotic spindle assembly
0 0.5 0.1 0.15 0.2
GeneRatio
p.adjust
d
GOBP_MAIT_GBS
protein folding
regulation of hemopoiesis
myeloid cell differentiation
'de novo' protein folding
chaperone-mediated protein folding
regulation of leukocyte cell-cell adhesion
regulation of T cell activation
'de novo' post-translational protein folding
response to unfolded protein
response to topologically incorrect protein
chaperone cofactor-dependent protein refolding
myeloid cell homeostasis
regulation of alpha-beta T cell activation
erythrocyte differentiation
cellular response to heat
protein refolding
negative regulation of inclusion body assembly
regulation of inclusion body assembly
cellular response to interleukin-7
response to interleukin-7
0 0.05 0.1 0.15
GeneRatio
p.adjust
GOBP_MAIT_Recovery
lymphocyte differentiation
T cell differentiation
regulation of leukocyte differentiation
regulation of T cell activation
regulation of hemopoiesis
regulation of T cell differentiation
regulation of lymphocyte differentiation
regulation of T cell differentiation in thymus
T cell differentiation in thymus
morphogenesis of an endothelium
endothelial tube morphogenesis
0 0.1 0.2 0.3
GeneRatio
p.adjust
e
GOBP_Tgd_GBS
regulation of hemopoiesis
lymphocyte differentiation
T cell differentiation
regulation of leukocyte differentiation
regulation of T cell activation
regulation of T cell differentiation
regulation of lymphocyte differentiation
fat cell differentiation
negative regulation of cell development
alpha-beta T cell differentiation
negative regulation of leukocyte activation
alpha-beta T cell activation
SMAD protein signal transduction
CD4-positive, alpha-beta T cell differentiation
regulation of alpha-beta T cell activation
CD4-positive, alpha-beta T cell activation
negative regulation of T cell differentiation
p38MAPK cascade
regulation of CD4-positive, alpha-beta T cell differentiation
negative regulation of lymphocyte differentiation
0 0.05 0.1 0.15
GeneRatio
p.adjust
GOBP_Tgd_Recovery
regulation of nucleotide-binding oligomerization domain containing 2 signaling pathway
negative regulation of inclusion body assembly
positive regulation of tumor necrosis factor-mediated signaling pathway
regulation of nucleotide-binding domain, leucine rich repeat containing receptor signaling pathway
regulation of inclusion body assembly
regulation of microtubule nucleation
nucleotide-binding oligomerization domain containing 2 signaling pathway
regulation of mitotic spindle assembly
protein refolding
inclusion body assembly
nucleotide-binding domain, leucine rich repeat containing receptor signaling pathway
chaperone cofactor-dependent protein refolding
positive regulation of microtubule polymerization
positive regulation of erythrocyte differentiation
regulation of spindle assembly
negative regulation of signal transduction in absence of ligand
negative regulation of extrinsic apoptotic signaling pathway in absence of ligand
positive regulation of microtubule polymerization or depolymerization
'de novo' post-translational protein folding
regulation of mitotic spindle organization
0 0.25 0.5 0.75 1
GeneRatio
p.adjust

Supplementary Figure S3. Gene Ontology enrichment analysis of cytotoxic and innate-like lymphocyte populations.

a–b. Bar plots showing enriched Gene Ontology Biological Process pathways in cytotoxic $CD8^+$ T-cell and natural killer cell populations in the GBS sample relative to the recovery sample. **c–e.** Bar plots showing enriched Gene Ontology Biological Process pathways in additional cytotoxic $CD8^+$ T-cell, MAIT-cell, and γδ T-cell populations across the GBS and recovery samples. Adjusted P values indicate over-representation of Gene Ontology terms within the exploratory cell-level DEG lists. Because individual cells were not considered independent biological replicates, these enrichment results do not represent patient-level biological inference.

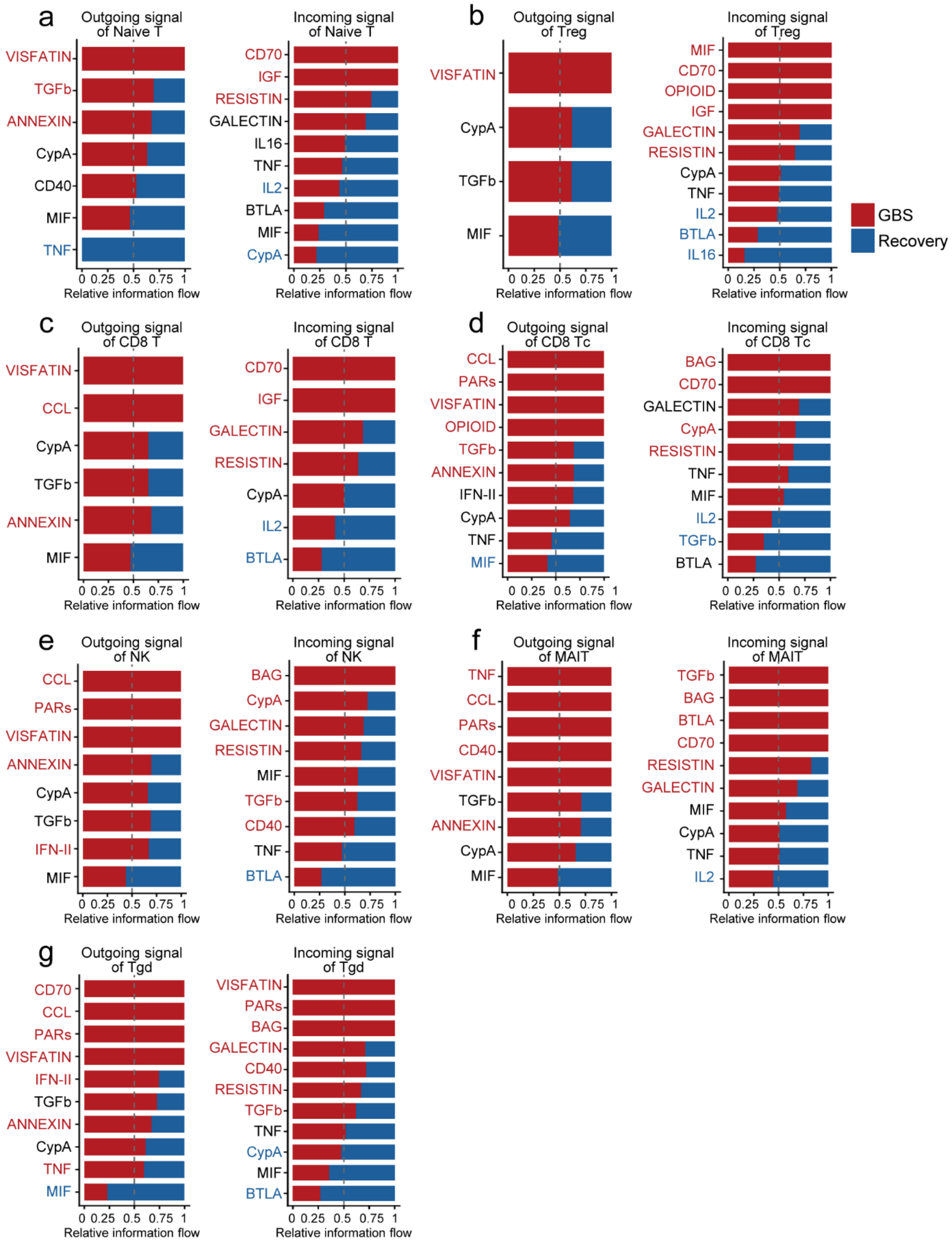

Supplementary Figure S4. Inferred cytokine-mediated communication patterns in T-cell and NK-cell populations.

a–g. Bar plots showing inferred outgoing and incoming cytokine-mediated signaling pathways in naïve T-cell subsets (Naïve_1 and Naïve_2), regulatory T cells, cytotoxic CD8^{+} T cells,

CD8$^+$ T cells, natural killer cells, MAIT cells, and γδ T cells in the GBS sample relative to the recovery sample. CellChat comparisons represent descriptive within-patient observations. CellChat P values, where applicable, reflect the package's internal assessment of individual ligand–receptor interactions within each sample and do not test differences between the two clinical time points. No patient-level inferential statistical test was performed.
